# Neurotoxicity and Oxidative Stress Development in Adult *Atya lanipes* Shrimp Exposed to Titanium Dioxide Nanoparticles

**DOI:** 10.3390/toxics11080694

**Published:** 2023-08-11

**Authors:** Stefani Cruz-Rosa, Omar Pérez-Reyes

**Affiliations:** Department of Environmental Sciences, College of Natural Sciences, University of Puerto Rico, Rio Piedras Campus, San Juan 00925, Puerto Rico

**Keywords:** acute exposure, catalase, effective concentration, macroinvertebrates, nanotoxicity

## Abstract

Titanium dioxide is a type of nanoparticle that is composed of one titanium atom and two oxygen atoms. One of its physicochemical activities is photolysis, which produces different reactive oxygen species (ROS). *Atya lanipes* shrimp affect detrital processing and illustrate the potential importance of diversity and nutrient availability to the rest of the food web. It is essential in removing sediments, which have an important role in preventing eutrophication. This study aimed to determine the toxic effect of changes in behavior and levels of oxidative stress due to exposure to titanium dioxide nanoparticles in *Atya lanipes* and to determine the effective concentration (EC50) for behavioral variables. The concentrations of TiO_2_ NPs tested were 0.0, 0.50, 1.0, 2.0, and 3.0 mg/L with the positive controls given 100 µg/L of titanium and 3.0 mg/L of TiO_2_ NPs ± 100 µg/L of titanium. After 24 h of exposure, significant hypoactivity was documented. The EC50 was determined to be a concentration of 0.14 mg/L. After the exposure to 10 mg/L of TiO_2_ NPs, oxidative stress in gastrointestinal and nervous tissues was documented. The toxic effects of this emerging aquatic pollutant in acute exposure conditions were characterized by sublethal effects such as behavior changes and oxidative stress.

## 1. Introduction

Titanium dioxide is a type of nanoparticle that is composed of one titanium atom and two oxygen atoms. One of its physicochemical activities is photolysis, which produces different reactive oxygen species (ROS) [1,2]. In this way, it is a chemically inert and photocatalytic nanoparticle that reflects all the colors of the light spectrum (since it reflects white light). Also, titanium dioxide is a white and fine powder [3]; it can exist in different forms, such as rutile, anatase, and brookite [4,5,6]. The anatase form is the most photocatalytic arrangement of this nanoparticle [7,8].

Due to their photocatalytic activities, titanium dioxide nanoparticles (TiO_2_ NPs) are of great interest because they are used in paints and coatings as self-cleaning, antimicrobial, and antifouling agents, a food additive, and as a UV absorber in cosmetics [9,10]. From 2006 to 2010, the commercial production of titanium dioxide was 5000 metric tons per year; from 2011 to 2014, it was more than 10,000 metric tons, and it is estimated to reach 2.5 million metric tons by 2025 [11].

The rapid production and use of TiO_2_ NPs result in a direct and indirect release into aquatic environments through bathing, industrial effluent, and engineering applications [12,13]. In this way, one of the ecosystems affected by the presence of TiO_2_ NPs is the freshwater ecosystem. The environmental concentrations of these NPs in freshwater ecosystems are variable in the scientific literature. However, the health of the river can be affected by TiO_2_ NPs in ways that are not yet quantified. Nevertheless, some studies have demonstrated the presence of TiO_2_ NPs in high concentrations in natural surface waters and rivers [14]. However, most studies focus on determining TiO_2_ NP levels in surface waters, and few studies have studied these in sediments. Despite the lack of standardized quantification of TiO_2_ NPs in aquatic ecosystems, European studies have determined that the concentrations of TiO_2_ NPs in freshwater range from 0.015 to 24.5 micrograms/liter [15]. On the other hand, studies have documented that soil concentrations may exceed 100 micrograms/liter [14]. However, concentrations of titanium (including TiO_2_ NPs) were measured in the United States and Canada in a study involving 15 rivers. The concentrations of titanium in these rivers ranged from 0.5 to15 micrograms/liter and, in some soils and sediments, about 10–100 g/kg with an average of less than 5 g/kg [16].

In the Caribbean and Puerto Rican freshwater ecosystems, we have an important shrimp species named *Atya lanipes* Holthuis 1963. *Atya lanipes* are a scraper/filter feeder shrimp that live part of their life as planktonic organisms until they reach the youth stage. This amphidromous life cycle of the Caribbean freshwater shrimp represents an important relationship between the headwaters and estuaries [17]. Crowl (2001) [18], among others, demonstrated that *Atya lanipes* affect detrital processing and illustrate the potential importance of diversity and nutrient availability to the rest of the food web. Also, *Atya lanipes* are essential in removing sediments from streams, which have an important role in preventing eutrophication in this aquatic environment. For this reason, any contaminant that can both settle or accumulate/dissolve in the water could harm this species and alter its role in the ecosystem. Therefore, TiO_2_ NPs, like other hydrophobic nanomaterials, tend to sediment as their fate in aquatic ecosystems. In this way, *Atya lanipes* will be susceptible to the presence of these engineering nanoparticles in two different aquatic ecosystems: estuaries and rivers. This makes *Atya lanipes* an excellent nano-toxicological model.

Studies with TiO_2_ NPs have found that these NPs, acting as adsorptive agents, interact with metals and are stored in sediments [19]. In addition, it has been shown that that adsorptive ability of TiO_2_ NPs can exacerbate the bioavailability and neurotoxicity of organic pollutants and pesticides [19]. There have also been several studies that focused on using the photocatalysis (formation of oxidative agents) of TiO_2_ NPs for remediation and treatment of wastewater but many of these studies did not consider the possible toxicity to aquatic organisms [20,21].

Researchers have recognized the reaction between nanomaterials and biological systems in many ecotoxicology studies [22]. However, most of these studies have been conducted in bacteria, cell lines, and rodent animals [23]. Nevertheless, these studies reveal the development of oxidative stress as the principal biological effect. Oxidative stress is produced by free radicals, which contain one unpaired electron and are highly reactive and capable of damaging molecules and transforming them into reactive molecules. These produce a redox chain reaction that damages cells and tissues in biological systems. This includes cell toxicity by oxidation of lipids, proteins, carbohydrates, and nucleotides leading to the formation of intracellular aggregates, mitochondrial dysfunction, excitotoxicity, and apoptosis. All biological systems have an antioxidant defense to avoid oxidative stress. The antioxidants act by decreasing the concentration of oxidants, preventing the initiation of the chain reaction by “sweeping” (covering or stopping a very highly reactive chemical) the first free radicals to form, binding to metal ions to prevent the formation of reactive species, transforming peroxides into less reactive products, and stopping the spread and increase in free radicals. One example of a very important antioxidant enzyme is catalase which acts as a metabolizer, transforming peroxides into H_2_O and O_2_. In this way, this enzyme minimizes oxidative stress damage [24].

The biological effects from oxidative stress induced by TiO_2_ NPs are known as dose-dependent toxicity indices. Some studies focused on green algae (*Desmodesmus subspicatus*) have determined that EC50 = 44 mg/L [25]. There are no dose–response toxicological analyses on *Atya lanipes* shrimp for behavioral variables (another important sublethal effect). These indices are imperative to explore in this species due to its susceptibility to contamination in water bodies, particularly for nanomaterials, as we have previously discussed. It is necessary to determine the toxicological indices for TiO_2_ NPs in terms of behavioral variables to analyze the environmental risk from this type of nanoparticles in the freshwater ecosystems inhabited by this species [23]. Also, this shrimp needs a healthy nervous system to complete its complex life cycle (which includes migrations), acquire food, avoid predation, and fulfill its other ecological roles.

This study aimed to determine the toxic effects of exposure to titanium dioxide nanoparticles reflected in changes in behavior and development of oxidative stress during the adult life cycle of *Atya lanipes* shrimp and to determine the dose–response index (EC50), for behavioral variables after exposure to different TiO_2_ NP suspension concentrations. We found that acute exposure to TiO_2_ NPs produced a toxic effect on the nervous system of *Atya lanipes* shrimp, resulting in hypoactivity as has been determined in the larval stage of this species [26]; we also observed the effects of TiO_2_ NPs at concentrations at the mg/L scale due to the capacity of anatase form of this nanoparticle to produce oxidative stress. A Probit analysis for behavioral variables showed an EC50 of TiO_2_ NPs at a low concentration. Also, the development of oxidative stress was evident and remained constant during the acute exposure, reflected in an increase in catalase enzyme activity.

## 2. Materials and Methods

### 2.1. Characterization of Titanium Dioxide Nanoparticles Suspensions (TiO_2_ NPs)

**Figure 1:**
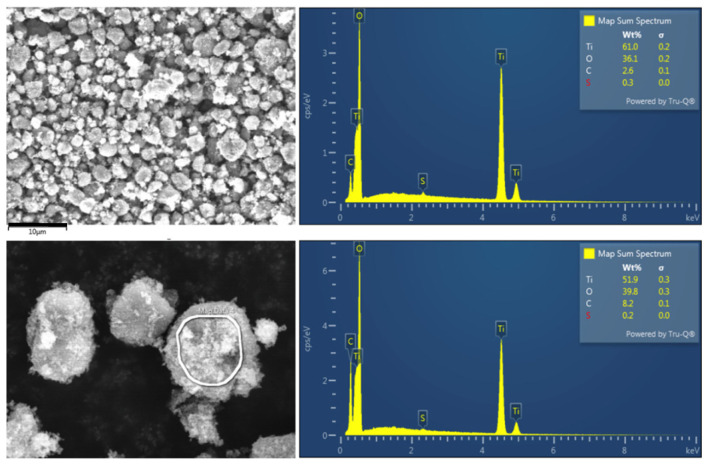
Elemental analysis through energy dispersive spectrum. The composition of the nanopowder of titanium dioxide in the analyzed sample demonstrates 61.0 wt% titanium, 36.1 wt% oxygen, 2.6 wt% carbon and 0.3 wt% sulfur.

Titanium dioxide nanopowder, with a particle size until 25 nm (Sigma Aldrich Chemical Company St. Louis, MO, USA, Titanium IV Oxide, Anatase nanopowder), was used to conduct the experiments. The titanium dioxide powder was spread on a weighing paper and gently picked up by the sticky carbon surface above the aluminum stubs. An S4700 II cFEG SEM (Hitachi High Technologies-America, Schaumburg, IL, US) with a silicon drift EDX detector (Oxford Instruments, X-MaxN, Abingdon, UK) was used to measure the surface morphology, elemental composition, and distribution of elements. All the SEM data reported were obtained at an acceleration voltage of 10 kV, and the images were collected with a Secondary Electron detector. The elemental mapping and energy spectrums were acquired with Aztec tools (Oxford Instruments, UK). The elemental analysis through the energy dispersive spectrum indicated the presence of Ti, O, C, and S elements (61, 36.1, 2.6, and 0.3 wt%, respectively) (Figure 1).

### 2.2. Atya lanipes Specimens Collection

Adult *Atya lanipes* specimens were collected and identified [27], using baited minnow traps at the Sonadora Stream at the El Verde Field Station, Rio Grande, Puerto Rico. The pools at the Sonadora Stream have a depth that range between 0.5 and 1.5 m, but we selected pools with a depth of 0.5 m. The sampling period was from January 2022 to June 2022 when the climate and specimen availability were favorable. Baited traps (dry cat food was used as the bait) were set in different pools along the stream and removed 24 h later. The collected shrimp were transported to the laboratory in a cooler under constant aeration. The shrimp were transferred individually to glass tanks (15 cm × 15 cm × 15 cm) with 1 L of dechlorinated water and constant aeration. The animals were acclimated for 72–120 h (three to five days) in the laboratory environment before the bioassay.

### 2.3. The Microcosm

**Figure 2:**
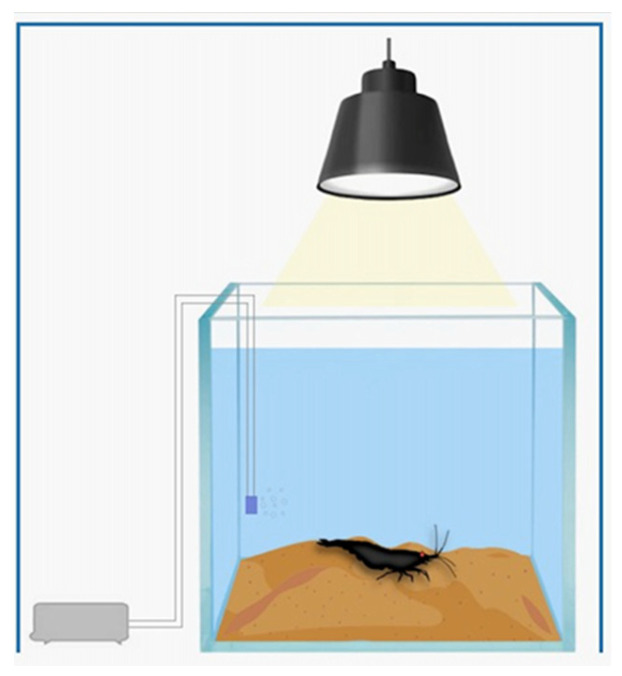
Microcosm: aquarium with 1 L of water, 150 g of artificial sediment, air stone connected to the air pump, light, and one *Atya lanipes* adult individual.

Fifty microcosms (25 for the control and 25 for each treated group) were designed. They consisted of a square aquarium containing 1 L of dechlorinated water; 150 g of synthetic sediment that was previously sterilized with activated carbon and heated in an oven at a temperature of 50–60 °C; an air stone with an air pump; and LED lamps with a 10 h/14 h light/dark photoperiod (controlled by an automatic timer). The water temperature was kept between 19 and 21 °C (Figure 2). These variables reflect the environmental conditions in which *Atya lanipes* live in the wild.

### 2.4. Acute Toxicity Tests

Before the bioassay, we mixed the titanium dioxide nanopowder with one L of dechlorinated and oxygenated (for 24 h) water using a magnetic stirrer at maximum speed for 30 min. Subsequently, TiO_2_ NPs were weighed to obtain final concentrations of 0.0, 0.50, 1.0, 2.0, and 3.0 mg/L. The nanoparticles were added into the fish tanks and left to fall by gravity; the aquarium water was mixed with a glass stirrer to obtain homogeneity in the microcosm. For each treatment, we set up 25 replicates each for the treated group and controls. The positive controls were exposed to titanium (100 mg/L) and TiO_2_ NPs and titanium (3.0 mg/L; 100 mg/L). The microcosms were set in an exposure bench covered with a piece of blue fabric to prevent the entry of external light and to control any possible contamination. Physicochemical parameters (temperature, salinity, dissolved oxygen, dissolved solids, and pH) of the water of each microcosm were taken before and after the exposure period using a Hanna HI98129 and Sper Scientific 850045 dissolved oxygen meter pen. After preparing the microcosms with their respective treatments, the *Atya lanipes* adults were randomly assigned to each treatment. Measurements of the post-orbital and cephalothorax of the shrimp were collected. Then, shrimp were individually introduced into the microcosms and left exposed to the treatments for an acute exposure of 24 h. No separation by sex was made because this species does not present clear sexual dimorphism traits. Instead, we randomly selected specimens ensuring a similar range of sizes for each treated and control group. Gravid shrimp were excluded from the bioassay. Shrimp were not fed during the bioassay.

### 2.5. Behavioral Analysis

A movement analysis was carried out after acute exposure to TiO_2_ NPs for 24 h. *Atya lanipes* shrimp are more active at night; therefore, in order to analyze these patterns in the laboratory setting, we created a recording scenario that simulates nighttime for these organisms. The shrimp recordings were performed in a red box with red light because they do not detect this light wavelength. Also, in control organisms, it was observed that, in the box, they were active at the corners and never went toward the center of the recording box. Moving to the center of the box has the possibility of being preyed on.

**Figure 3:**
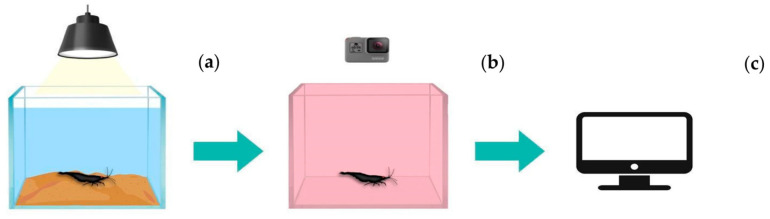
Movement analysis performed on *Atya lanipes* exposed to different concentrations of TiO_2_ NPs for 24 h. (**a**) The microcosm prepared for the bioassay with the *Atya lanipes* shrimp exposed to the nanoparticles; (**b**) the acclimatization process of the shrimp in the recording area inside a red plastic box; (**c**) the analysis of the movement in the videos using Loligo Systems^®^ 5th version software.

Each specimen of *Atya lanipes* in the experiment was removed from the exposure tank and acclimatized for 3 min in a red plastic box with 2 L of dechlorinated water that was previously oxygenated for 24 h (it was changed with new water for each shrimp analyzed to avoid contamination and to minimize errors in the analysis). The shrimp were set in the red box with new water because the artificial sediment alters the video, creating artifacts that result in errors in the video; in addition, the movement of the TiO_2_ NPs in the water to the red box will result in the displacement of the nanoparticles that were set in the sediment. This acclimatization was performed in the recording room under total darkness and with no sound. After the acclimation period, the movement of the shrimp in the red tank was recorded for 5 min using a camera (Go Pro Hero 6^®^, GoPro, Inc., San Mateo, CA, USA). These visual patterns are analyzed from the recording and, through Loligo Systems^©^ 5th version software (Loligo Systems, Viborg, Denmark), we quantified movement variables such as distance traveled and active time, among others (Figure 3).

### 2.6. Oxidative Stress Analysis

**Figure 4:**
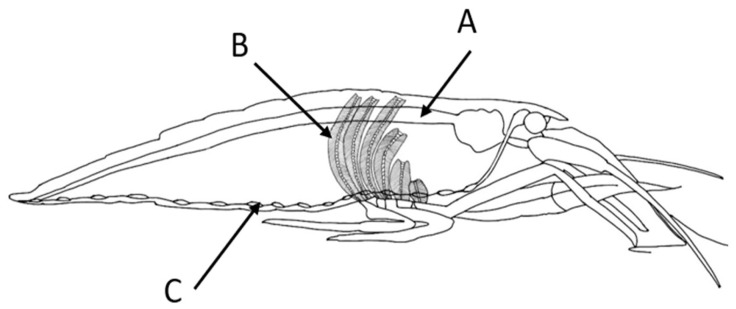
Diagram of *Atya lanipes* shrimp and the anatomy of the dissected tissues for the oxidative stress assessment. (**A**) Gut, (**B**) gills, and (**C**) nerve cord.

To analyze oxidative stress from 0 to 11 days after an exposure in *Atya lanipes*, we dissected five specimens that had been previously exposed to each TiO_2_ NPs concentration and the control treatments. These shrimps were dissected every two days. The shrimp exposed to the nanoparticles were not fed during the 11 days. The *Atya lanipes* specimens were preserved by gradually putting the shrimp at a cold temperature to decrease their biological activity without causing strong stress that would alter our results. This was performed in approximately five minutes; then, we removed the gastrointestinal tract (gut), the ventral nervous system (nerve cord), and the gills (Figure 4).

We used the Catalase (CAT) Activity Assay kit (Catalog No: MBS2540413; colorimetric method; sensitivity 0.27 U/mL) to determine the catalase enzymatic activity in each tissue sample for each period. Then, we calculated the enzymatic activity in U/mgprot with the formula CAT activity = ΔA × 32.5/1 × V × f/Cpr where 32.5 is the reciprocal of the slope, 1 is the reaction time, ΔA is the ODcontrol—ODsample, V is the volume of the sample (mL), f is the l factor of the sample before the test, and Cpr is the concentration of protein in the sample (g/L).

### 2.7. Statistical Analyses

Parametric statistical approach was performed because of the normality of the data. Descriptive statistics were used to summarize the values of the physicochemical parameters. To compare the physicochemical parameters among treatments and control, one-way ANOVA was conducted. The shrimp movement was compared among treatments by a one-way ANOVA and Tukey tests. A Probit analysis was used to compare the toxicity levels in the movements. Lastly, we used the formula suggested by the catalase kit to analyze the development of oxidative stress. All the descriptive and statistical analyses were performed using Minitab 17 Statistical Software [28].

## 3. Results

### 3.1. Specimens of Atya lanipes and Physicochemical Parameters

The sample of 25 organisms for the control and exposure groups was chosen to normalize the average shrimp size to standardize the bioassay and obtain reliable data. Cephalothorax lengths (CL) of the control and TiO_2_ NP-exposed groups ranged from 14.6 to 16.9 mm on average, and the post-orbital lengths (POL) were between 11.3 and 13.9 mm on average.

To maintain the internal environmental conditions of the microcosms stable during the bioassay, we measured the physicochemical parameters of the water before and after the 24 h exposure to TiO_2_ NPs. The temperature of the microcosm was the same as laboratory temperature which varied between 19 and 21 °C for the entire exposure period (Table 1).

The one-way ANOVA for the physicochemical properties showed no significant differences between any pre- and post-exposure time variable. No changes in pH, conductivity, or salinity measurements before and after exposure were observed. However, dissolved the oxygen measurements showed a decrease in groups exposed to TiO_2_ NPs; this result was not observed in the controls. An increase in dissolved oxygen concentrations was observed after 24 h of exposure. This was expected due to the presence of the constant oxygen pump in each microcosm during the entire exposure period.

### 3.2. Movement Assessment

**Figure 5:**
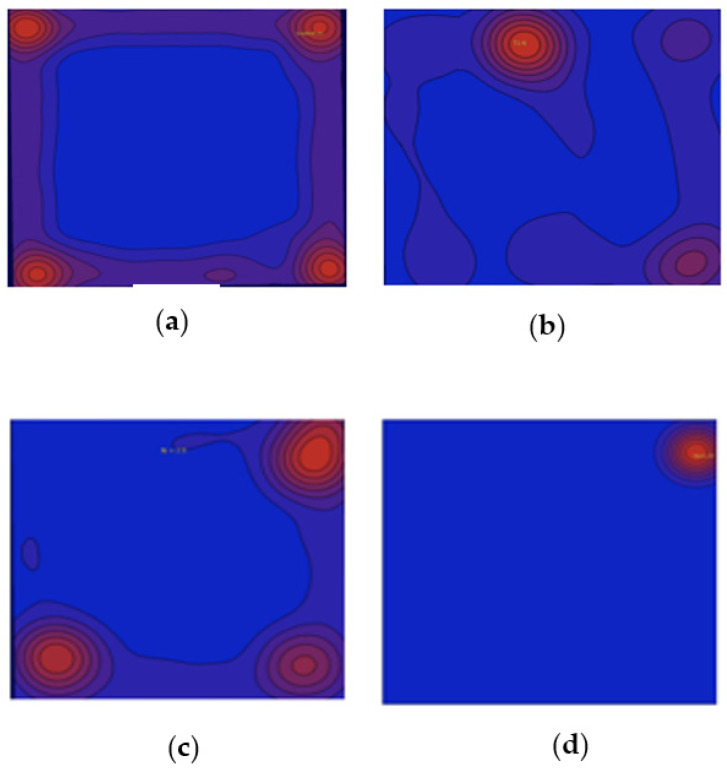
Heat maps for the adult *Atya lanipes* shrimp behavior in all treatment and control groups. Red areas = more time spent in that area by the shrimp. (**a**) Control, (**b**) titanium (100 micrograms/mL), (**c**) titanium (100 micrograms/mL) ± TiO_2_ NPs (3 mg/L), (**d**) TiO_2_ NPs (3 mg/L).

The analysis of movement after the exposure to TiO_2_ NPs for 24 h showed significant changes leading to hypoactivity. The heat maps for the adult shrimp in the control group (0.0 mg/L of TiO_2_ NPs) showed a preference for the corner of the box over any other location. Their movements were limited to the corners of the box. In contrast, the exposure group showed erratic preferences and less exploration movements, especially in the TiO_2_ NP-exposed groups (Figure 5).

**Figure 6:**
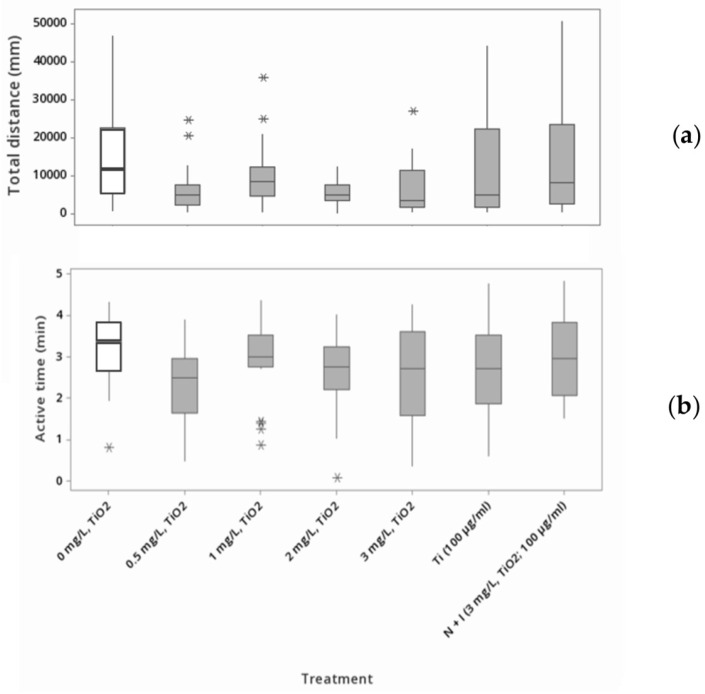
Assessment of movement of adult *Atya lanipes* shrimp (*n* = 25 for each treatment). (**a**) Total distance moved (mm) and (**b**) active time (min) of *Atya lanipes* shrimp after 24 h acute exposure to different TiO_2_ NP suspension concentrations, titanium (100 mg/L), and TiO_2_ NPs + titanium (3 mg/L; 100 mg/L). * = outliers.

This hypoactivity characteristic in the movement assessment was statistically evaluated using one-way ANOVA with a Tukey test. During the 24 h of exposure, we observed significant differences between the exposed and control groups in the total distance moved and active time (min) (*p* < 0.05) (Figure 6). The average of the total distance (*n* = 25) traveled by the adult shrimp in the negative control group was 15,372.6 mm ±/−2581.8 mm with a minimum of 592.1 mm and a maximum of 46,759.8 mm during the 24 h of exposure. Also, for the positive controls exposed to titanium, they moved a total distance of 21,039.8 mm ± 10,070.0 mm with a range of 403.7 mm to 254,938.2 mm on average. In the second positive control (titanium ± TiO_2_ NPs), the average total distance was 16,525.2 mm ± 4188.4 mm with a range of 272.5 mm to 95,032.1 mm. For the different treatments of TiO_2_ NPs, (0.5, 1.0, 2.0, and 3.0 mg/L), we observed a total distance average of 6072.7 ± 1150.8, 9644.3 ±1585.3, 5429.7 ± 626.0, and 6571.0 ± 1388.9 mm, respectively. Moreover, the total distance moved for the exposed shrimp ranged from 7.1 mm to 35,659.7 mm. The one-way ANOVA for the comparison of the total distance moved of the adult *Atya lanipes* shrimp in the control groups and the exposure groups showed a significant difference (F (6167) = 2.6; *p* < 0.05) among groups.

The active time (min) of the adult shrimp (*n* = 25) was 3.18 min ± 0.17 min with a minimum of 0.8 min and a maximum of 4.32 min for the negative control during the 24 h of exposure. Consequently, the active time for the positive controls was 2.74 min ± 0.22 min and 2.98 min ± 0.19 min with minimums of 0.6 and 1.51 and maximums of 4.77 and 4.84 min, respectively. The exposure groups’ (TiO_2_ NP suspension concentrations of 0.5, 1.0, 2.0, and 3.0 mg/L) average active time were 2.37 ± 0.18, 2.96 ± 0.18, 2.62 ± 0.17, and 2.54 ± 0.22 min. We observed minimums of 0.47, 0.85, 0.06, and 0.35 min with maximums of 3.90, 4.37, 4.02, and 4.26 min for the TiO_2_ NP suspension concentrations of 0.5, 1.0, 2.0, and 3.0 mg/L, respectively. The one-way ANOVA for the comparison of all TiO_2_ NP suspension concentrations and the positive and negative controls for the active time variable after 24 h of exposure showed a significant difference (F (6167) = 2.24; *p* < 0.05) between groups. The Tukey test analysis showed values of *p* < 0.05 for the treatment of 0.5 mg/L TiO_2_ NPs.

For the movement variable of active time, a Probit analysis was conducted. The lognormal probability plot at a 95% CI (−6.320–2.424) showed a coefficient of 0.20 for the different concentrations, standard error = 0.19, Z= 1.03, and a *p* value = 0.30. Also, the regression data showed a chi-square = 0.025, Df = 2, and Pearson = 0.99. The EC values for 50, 60, 70, and 90% of events are presented in Table 2.

### 3.3. Oxidative Stress Assessment

The development of oxidative stress, defined as increased catalase enzyme activity, in shrimp exposed to TiO_2_ NPs was compared against that of the unexposed shrimp. The branchial, nervous, and gastrointestinal tissues were obtained from five shrimp for each analysis time and for each nanoparticle suspension concentration and control group, after 24 to 264 h of exposure. The average environmental conditions of the microcosms of the shrimp used to obtain the fresh tissue samples are summarized in Table 3. During the 0–264 h exposure to TiO_2_ NPs, the temperature varied between 18.5 and 19.9. The pH ranged from 5.6 to 7.7. A pH below 6.0 was seen during 216 to 264 h of exposure. The conductivity varied from 0.3 to 0.5 µs. Dissolved oxygen during the exposure ranged from 7.8 to 9.2 mg/L and the salinity from 0.1 to 0.2 ppm.

The specimen sizes were determined by measuring their POL and CL lengths (Table 4). The POS lengths of the samples ranged from 10 to 17 mm and the CEF from 11 to 22 mm. The average POS lengths of the *Atya lanipes* specimens were 12.7 ± 1.3, 13.3 ± 1.3, 12.1 ± 1.0, 11.1 ± 0.2, 12.2 ± 1.0, 12.0 ± 1.0, and 10.8 ±/−0.2 mm for the control, 24 h, 72 h, 120 h, 168 h, 216 h, and 264 h exposure specimens, respectively, and the average CEF lengths were 14.3 ± 2.0, 16.6 ± 1.4, 14.6 ± 1.4, 13.6 ± 0.2, 14.2 ± 1.1, 14.9 ± 1.2, and 14.0 ± 0.4 mm, respectively. The POS and CEF measurements for the control and treated groups were not significant different.

Catalase activity in gastrointestinal tissues between 24 and 264 h of exposure to 10 mg/L of TiO_2_ NPs was accessed. The average catalytic activity of catalase in the control group was 10.97 ±/−0.2 U/mg prot. The catalase activity at each exposure time (24, 72, 120, 168, 216, and 264 h) was 0.79, 15.93, 27.94, 55.90, 19.5, and 17.77 U/mg prot, respectively. After 24 h of exposure, the catalase activity was approximately 0, which indicates a state of extreme shock and oxidative stress development in a short period. However, after 264 h of exposure, there was higher catalase enzymatic activity compared to the control group.

The analysis of catalase activity in the gill tissues showed that, after 24 h of exposure, the enzyme activity was significantly higher in the exposure group (54.38 U/mgprot) than the control group (18.09 ±/−0.2 U/mg prot). This result differed from the values we obtained in the analysis of catalase activity in the gastrointestinal tissues. In this case, the enzymatic activity was higher after 24 h but after 72 h (22.48 U/mg prot), 120 h (28.87 U/mg prot), 216 h (22.60 U/mg prot), and 264 h (20.39 U/mg prot), the enzyme activity was similar to that of the control group, except for the exposure time of 168 h (104.73 U/mg prot) when the enzyme activity was higher than that after 24 h of exposure.

**Figure 7:**
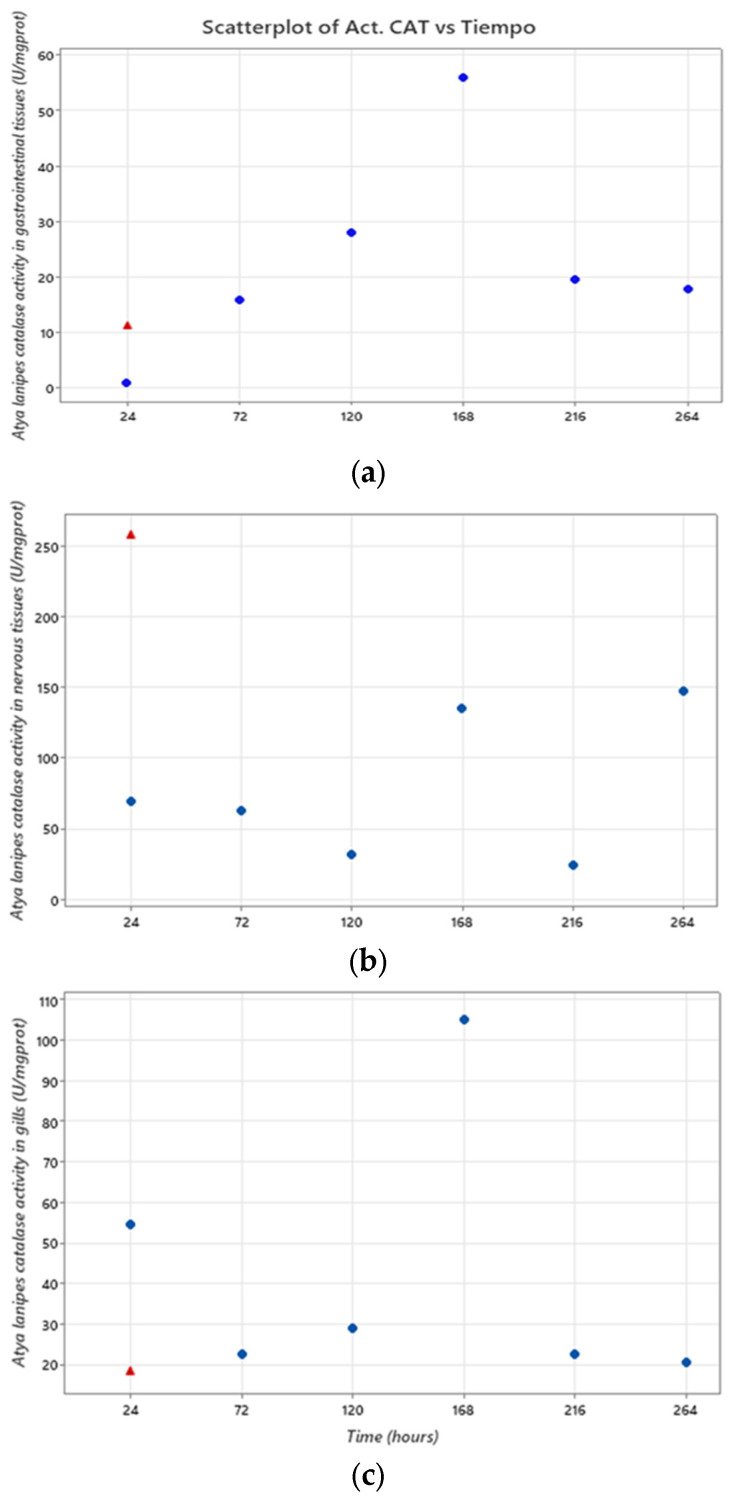
*Atya lanipes* catalase activity in tissues after 24 to 264 h of exposure to 10 mg/L of TiO_2_ NPs. The red triangle is the catalase activity in the control group. (**a**) Gastrointestinal tissues, (**b**) nervous tissues, and (**c**) gill tissues.

In the nervous tissues from the control group, the average enzyme activity was 258.04 ±/−0.3 U/mg prot. However, the catalase activity for each exposure time was 69.44, 62.23, 31.70, 135.0, 24.01, and 147.02 U/mg prot for the exposures times of 24, 72, 120, 168, 216, and 264 h, respectively (Figure 7).

## 4. Discussion

This study assessed the neurotoxicity of TiO_2_ NPs in *Atya lanipes* shrimp after a 24 h acute exposure and documented the development of oxidative stress after 24 to 264 h of exposure. Currently, more studies must be carried out related to the biocompatibility and toxicity of engineering nanomaterials [29,30]. This is because their use continues to increase in many sectors globally including medicine, the food industry, and technology [31]. The biological and ecosystem interactions with nanomaterials are important in defining and preventing future impacts on ecosystems such as freshwater ones. Also, these data could help in the production, management, and future environmental regulation of engineered nanoparticles. Therefore, their possible interactions with living organisms and their toxicity must be considered before commercial use.

Most of the nano-toxicological studies have been carried out in organisms that are not exposed to nanomaterials in the aquatic environment where they naturally live [32]. However, in the communities and populations of freshwater shrimp, there has been little attention on this matter. It has been shown in studies related to other aquatic contaminants such as pesticides, that these macroinvertebrates are a good toxicological model [33]. For *Atya lanipes* and TiO_2_ NPs, their lethal and sublethal effects in larval stages [26] have been evaluated and documented. A significant hypoactivity in the movement of the larvae after acute exposure was observed. In this study, in the adult stage of the *Atya lanipes* shrimp, we observed that, after a 24 h acute exposure to low concentrations of TiO_2_ NPs, the shrimp exhibited hypoactive movements. Significantly, the heat maps documenting the “normal” movements of exploration of the *Atya lanipes* shrimp to be in the corners of the box rather than the center. This behavior is expected in this shrimp species since it is a standard mechanism to remain still or in the center to evade predation. Thus, when not exposed to any treatment, healthy adult shrimp showed constant movement in this pattern for more than half of the recording time. However, shrimp exposed to titanium treatment or titanium and titanium dioxide nanoparticles began to show a decrease in this pattern of exploration. In particular, the shrimp exposed to 3 mg/L TiO_2_ NPs did not show movement within the recording box. This is consistent with other studies that evaluated animal behavior after exposure to this nanomaterial, as neotropical tadpoles and zebrafish also presented this characteristic hypoactivity [34,35].

Scientific investigations have shown the relevance of the sublethal effects of toxicants. In the past, we referred to toxicity primarily as the ability of contaminants to cause the death of the organisms under study. Today, we know that sublethal effects can produce a great disparity in the organism’s functioning that can be extrapolated to effects on the ecosystem it is part of. Therefore, it is necessary to know the probability that a specific contaminant concentration will produce a defined effect, in this case, a sublethal effect. When carrying out the Probit analysis for the variable of active time of the shrimp, we obtained an average index of 0.14 mg/L. These data demonstrate the susceptibility of this species to an acute exposure to TiO_2_ NPs of only 24 h. The need to evaluate toxicological indices for sublethal effects for this species at different exposure times is evident.

In general, acute toxicity studies (≤96 h) in bacteria (*V. fischeri*), green algae (*Pseudokirchneriella* sub-capitata and *Chydorus sphaericus*), some crustaceans (*D. magna* and *T. platyurus*), and fish embryos (*D. rerio*) have shown little or no toxicity when exposed to TiO_2_ NPs [36,37,38]. Recent research shows that exposure to many emerging contaminants can induce biochemical responses such as oxidative stress (as an example of sublethal effects) after an acute exposure without any lethality for that same exposure time [39]. However, a recent study evaluated the nanotoxicity of TiO_2_ toward the freshwater shrimp *Atya lanipes* in their zoea larval stages and the results showed that exposure to these nanoparticles induced mortality after 48 h of exposure, with edema, less pigmentation development, and hypoactivity in the larvae [26].

The characteristic biological effect of TiO_2_ NPs is the development of oxidative stress. Oxidative stress occurs when reactive oxygen species (ROS) are produced uncontrollably [24]. ROS are interrelated with many cellular mechanisms. They are highly reactive species which allows them to react with many biomolecules, affecting their function and/or damaging them and potentially leading to cellular apoptosis [40,41]. Fortunately, the cell has molecules and enzymes that have antioxidant roles. One of the most important is the enzyme catalase. The activity of this enzyme below or above the expected or normal values may indicate an increase in ROS and therefore the presence of oxidative stress [42]. This excess ROS in response to a xenobiotic contaminant can overwhelm the antioxidant mechanisms present in the organism [43]. This can cause oxidative damage and loss of compensatory mechanisms and a suppression of antioxidant enzymatic activities [43,44]. In this study, the catalase enzymatic activity of the groups exposed to TiO_2_ NPs were significantly different from that of the control group. It was shown that a 24 h acute exposure to TiO_2_ NPs resulted in the development of oxidative stress in gill, gastrointestinal, and nervous tissues.

The neurotoxicity after a 24 h acute exposure to TiO_2_ NPs and the development of oxidative stress in the freshwater shrimp species *Atya lanipes* was documented in this study. The toxic effects of acute exposure to this emerging aquatic pollutant was characterized by sublethal effects such as behavioral changes and induction of oxidative stress. Also, we suggest that freshwater shrimp are an excellent nano-toxicological model because of their life cycle and susceptibility to the presence of nanomaterials in their freshwater ecosystems and their ecological role in the biofiltration of natural organic particles in these ecosystems [45,46,47]. Future research should evaluate other sublethal effects such as bioaccumulation of the TiO_2_ NPs in *Atya lanipes* shrimp to understand their biological routes.

Some limitations of this study are in the assessment of the movement of the shrimp when exposed to TiO_2_ NPs. It was not possible to record the shrimp in the same exposure tank due to the presence of artificial sediment that was part of the microcosm and because the system that was used to analyze the behavior of the shrimp did not recognize the sediment as different from the shrimp due to their similar dark colors. It was not possible to determine “locomotion” so the “movement” of the shrimps was analyzed. Although it was recorded in a red box with red lights, which cannot be detected by these shrimps, and we promoted their normal active behavior at night, the removal of the shrimp from the exposure microcosm to fresh, clean, and oxygenated water prevented us from determining their locomotion. However, a short acclimatization period was allowed to avoid losing the effect of the TiO_2_ NPs in the exposure medium. All other variables were successfully controlled to obtain a movement assessment after exposure to TiO_2_ NPs.

This research contributes to our understanding of the nanotoxicity of TiO_2_ NPs and provides a starting point to determine the importance of regulating this type of nanomaterial and controlling concentrations of this contaminant in freshwater ecosystems. These results are very relevant in the scientific community because they present data for an area that has not received much attention in the field of nano-ecotoxicology and can promote research towards understanding the biocompatibility of these nanomaterials. At the same time, it shows us the need to prevent biological, ecological, and environmental impacts in the short, medium, and long term, for the purpose of conserving aquatic environments and biodiversity both in Puerto Rico and around the world.

## Figures and Tables

**Table 1:** Average (±standard error, SE) of the microcosm’s chemical parameters during the assay (pre- and post-exposure to TiO_2_ NPs for 24 h) for each control and TiO_2_ NP suspension concentrations.

Group	Time	pH + S.E.	Conductivity + S.E. (ms)	Salinity + S.E. (ppm)	O_2_, + S.E. (mg/L)
0.0 mg/L; TiO_2_ NPs	Pre	8.2 ± 0.04	328.2 ± 19.0	0.17 ± 0.01	8.7 ± 0.2
	Post	8.2 ± 0.04	341.5 ± 15.0	0.17 ± 0.01	8.8 ± 0.1
0.50 mg/L; TiO_2_ NPs	Pre	7.8 ± 0.003	468.7 ± 1.0	0.23 ± 0.001	9.2 ± 0.02
	Post	7.7 ± 0.02	436.0 ± 9.1	0.22 ± 0.004	8.0 ± 0.1
1.0 mg/L; TiO_2_ NPs	Pre	8.3 ± 0.00	353.2 ± 0.8	0.23 ± 0.0001	9.3 ± 0.1
	Post	7.7 ± 0.02	362.8 ± 2.3	0.22 ± 0.0001	8.4 ± 0.1
2.0 mg/L; TiO_2_ NPs	Pre	7.8 ± 0.01	469.6 ± 3.6	0.23 ± 0.002	9.5 ± 0.1
	Post	7.7 ± 0.02	475.2 ± 4.2	0.24 ± 0.002	7.9 ± 0.1
3.0 mg/L; TiO_2_ NPs	Pre	8.2 ± 0.04	332.2 ± 17.3	0.17 ± 0.01	8.6 ± 0.1
	Post	8.1 ± 0.04	337.4 ± 16.2	0.17 ± 0.01	8.9 ± 0.1
Titanium (100 mg/L)	Pre	8.2 ± 0.04	332.4 ± 16.0	0.17 ± 0.01	8.6 ± 0.1
	Post	8.1 ± 0.04	367.2 ± 21.0	0.18 ± 0.01	8.9 ± 0.1
TiO_2_ NPs (3 mg/L) ± Titanium (100 mg/L)	Pre	8.2 ± 0.04	340.4 ± 21.0	0.17 ± 0.01	8.6 ± 0.1
	Post	8.1 ± 0.04	371.8 ± 22.4	0.19 ± 0.01	8.9 ± 0.1

**Table 2:** Effective concentration of TiO_2_ NPs (mg/L) for active time variable of *Atya lanipes* shrimp after an acute 24 h exposure.

*Point*	*Concentration (mg/L)*
	*24 h*
EC50	0.143
EC60	0.520
EC70	2.073
EC80	10.467
EC90	98.868

**Table 3:** Average ± S.E. of the microcosms’ chemical parameters during the bioassay for each exposure time (10 mg/L of TiO_2_ NPs after 24 to 264 h).

Time (Hours)	Temperature + S.E. (°C)	pH + S.E.	Conductivity + S.E. (µs)	DO + S.E. (mg/L)	Salinity + S.E. (ppm)
Control group	19.4 ± 0.01	7.0 ± 0.1	0.4 ± 0.01	8.9 ± 0.04	0.2 ± 0.01
24	19.9 ± 0.02	7.1 ± 0.2	0.3 ± 0.004	8.3 ± 0.1	0.2 ± 0.01
72	18.5 ± 0.1	6.1 ± 0.1	0.4 ± 0.01	9.0 ± 0.1	0.2 ± 0.01
120	18.8 ± 0.02	6.8 ± 0.1	0.4 ± 0.0	8.7 ± 0.2	0.2 ± 0.001
168	18.6 ± 0.02	7.1 ± 0.1	0.4 ± 0.0	7.8 ± 0.01	0.2 ± 0.0
216	19.2 ± 0.1	5.8 ± 0.1	0.4 ± 0.01	8.4 ± 0.1	0.2 ± 0.004
264	18.7 ± 0.1	6.0 ± 0.04	0.4 ± 0.001	8.9 ± 0.04	0.2 ± 0.01

**Table 4:** Average ± S.E. of the size of the dissected specimens at each exposure time presented as post-orbital and cephalothorax lengths.

Time of Exposure (Hours)	POS + S.E. (mm)	CEF + S.E. (mm)
Control	12.7 ±/−1.3	14.3 ±/−2.0
24	13.3 ±/−1.3	16.6 ±/−1.4
72	12.1 ±/−1.0	14.6 ±/−1.4
120	11.1 ±/−0.2	13.6 ±/−0.2
168	12.2 ±/−1.0	14.2 ±/−1.1
216	12.0 ±/−1.0	14.9 ±/−1.2
264	10.8 ±/−0.2	14.0 ±/−0.4

## Data Availability

Not applicable.
